# A lactating woman presenting with puerperal pneumococcal mastitis: a case report

**DOI:** 10.1186/1752-1947-7-114

**Published:** 2013-04-25

**Authors:** Barbara Miedzybrodzki, Mark Miller

**Affiliations:** 1 Division of Internal Medicine, Jewish General Hospital, 3755 Cote-Ste-Catherine, Montreal, Quebec H3T 1E2, Canada; 2 Division of Infectious Diseases, Jewish General Hospital, 3755 Cote-Ste-Catherine, Montreal, Quebec, H3T 1E2, Canada

**Keywords:** Mastitis, Pneumococcal infection, Puerperal mastitis, *Streptococcus pneumoniae*, Pneumococcal vaccine

## Abstract

**Introduction:**

*Streptococcus pneumoniae* is an uncommon etiologic agent in soft-tissue infections.

**Case presentation:**

We report the case of a 35-year-old Caucasian woman who presented to our facility with puerperal pneumococcal mastitis, and review the only other three cases of pneumococcal mastitis described in the medical literature.

**Conclusions:**

The roles of the various pneumococcal vaccines in preventing this disease are discussed.

## Introduction

Puerperal mastitis occurs most commonly during the first three to six months post-partum in breastfeeding mothers. Up to 25 percent of breastfeeding women have experienced at least one episode of mastitis, and recurrent mastitis has been reported in four to eight-and-a-half percent of breastfeeding women [1]. The most common causative organism of mastitis is *Staphylococcus aureus*. Other less common organisms include coagulase-negative staphylococci*,* beta-hemolytic streptococci (Lancefield groups A or B)*, Escherichia coli*, and *Corynebacterium* species [2]. *Streptococcus pneumoniae* is an extremely rare cause of mastitis. In this paper, we present the case of a healthy 35-year-old woman who presented to our facility with puerperal pneumococcal mastitis, and review the only three other cases of pneumococcal mastitis described in the medical literature.

A literature review using a MEDLINE search from 1950 to July 2010 revealed only two cases of puerperal mastitis and one case of non-puerperal mastitis caused by *S. pneumoniae* (Table 1). The first case of pneumococcal mastitis was described by DiNubile *et al*. in 1991 in a 23-year-old woman with systemic lupus erythematosus who was being treated with prednisone but was not lactating [3]. She presented with an abscess of the left breast and the aspirate revealed *S. pneumoniae* and *Bacteroides fragilis.* The second case, described by Wüst *et al*. in 1995, was in a healthy 38-year-old woman breastfeeding her nine-month-old daughter [4]. In that case, serotyping was performed on a nasal and throat swab taken from the child as well as from the breast. All three cultures revealed *S. pneumoniae* serotype 6B, which the authors reported as being the second most frequent type found in the region at that time. The third case was published by Kragsbjerg *et al*. in 1995, concerning a 38-year-old woman who presented with purulent secretions from the breast while she was breastfeeding her four-month-old child [5]. Cultures taken from the breast and from the nasopharynx of the child revealed the same serotype of *S. pneumoniae.*

**Table 1:** Review of the literature of cases of pneumococcal mastitis

**First author, year and month published**	**Age of patient (years)**	**Time from last birth (months)**	**Localization of mastitis**	**Breast culture results (serotype)**	**Child's culture results**	**Treatment**
Present report	35	8	Right breast	*S. pneumoniae* type 19A	Not tested	Vancomycin and cefazolin followed by cefadroxil
*DiNubile, 1991	23	Non-puerperal	Left breast	Mixed: *S. pneumoniae* (type not reported) and *B. fragilis*	Not applicable	Cefazolin (initially)
Wüst, 1995 (February)	38	9	Left breast (lower medial quadrant)	*S. pneumoniae* (type not reported)	*S. pneumoniae* (type not reported)	Benzyl penicillin followed by phenoxymethylpenicillin
Kragsbjerg, 1995 (November)	38	4	Left breast (upper and lower lateral quadrants)	*S. pneumoniae* type 6B	*S. pneumoniae* type 6B	Flucloxacillin

*Non-puerperal mastitis.

## Case presentation

A 35-year-old Caucasian woman who was breastfeeding her eight-month-old twins presented to our facility with a three-day history of fronto-parietal headache, fever, general malaise, and two episodes of syncope on the day of admission. On further questioning, she also reported increasing pain in her right breast over the last 24 hours.

She appeared toxic and was febrile (39.0°C axillary temperature). A physical examination revealed an exquisitely tender right breast that was erythematous and indurated in the right lower lateral quadrant. There was, however, no area of fluctuance although purulent milky secretions could be expelled from the right nipple with mild peri-areolar pressure. These secretions were cultured. Slightly tender right axillary adenopathy was also present.

The results of laboratory investigations were unremarkable, including a normal blood count, except for the presence of a left shift with 80 percent neutrophils (total white blood cell count of 9.8×10^9^ cells/L). Several diagnostic investigations were performed, including a lumbar puncture, cerebral computed tomography (CT) and magnetic resonance imaging (MRI) scans, and blood cultures, all of which yielded normal results. A clinical diagnosis of puerperal mastitis was made, and treatment with intravenous vancomycin and cefazolin was initiated. Our patient continued pumping her breast milk. On the day after admission, increased amounts of pus were noted draining from the right nipple with each breast pumping. Our patient’s fever and rigors resolved within 48 hours. Culture of the breast secretions at the time of admission revealed heavy pure growth of *S. pneumoniae*, polysaccharide serotype 19A, which was susceptible to penicillin, cephalosporins, macrolides, tetracyclines and vancomycin. Her hospital course was uncomplicated and she was discharged home on day three post-admission with a 10-day course of oral cefadroxil. Neither of her babies showed any evidence of a respiratory tract infection prior to our patient’s illness; nasopharyngeal culture tests from the babies were not performed as they were at home with the father and unavailable for culture sampling.

## Discussion

Pneumococcal mastitis is an extremely rare entity and, to the best of our knowledge, there have been only three other case reports in the literature, two of which were puerperal. *S. pneumoniae* is a leading cause of respiratory tract infections and meningitis in both children and adults. It is, however, a rare cause of skin and soft-tissue infections and the cases reported are mostly described in patients who have some degree of immunosuppression [6]. Our patient, whose case we present here, was a healthy 35-year-old immunocompetent woman and there were no signs of any connective tissue diseases or other coincidental health issues.

Although neither of her babies showed any evidence of a respiratory tract infection prior to our patient’s illness, and testing of the babies was not undertaken due to their unavailability, it appears that the most probable way in which the mother became infected with *S. pneumoniae* serotype 19A was from one or both of the nasopharyngeal tracts of the babies during breastfeeding. In both of the previous case reports [4,5], the breastfed babies had tested positive on nasopharyngeal swabs and showed symptoms of mild respiratory tract infections, which is consistent with our interpretation of the mode of transmission of the *S. pneumoniae* in mastitis. Our patient's twin babies were both routinely vaccinated at two and four months of age with Prevnar-7® (Wyeth, Collegeville, PA, USA), which contains capsular antigens of *S. pneumoniae* serotypes 4, 6B, 9V, 14, 18C, 19F, and 23F. Thus, the serotype 19A *S. pneumoniae* isolated in our patient was not part of the seven-valent pneumococcal conjugate vaccine administered to children in the province of Quebec, where our patient resided at the time of her illness. Current Quebec immunization guidelines recommend vaccination of healthy babies with a pneumococcal seven-valent conjugate vaccine (Prevnar-7®) to be given in three doses administered at two, four and 12 months of age [7]. However, since the introduction of Prevnar-7®, there has been growing concern of the development and spread of the pneumococcal serotypes not covered in the vaccine. A recent review by Reinert *et al*. describes global indicators showing that serotype 19A is now the most prevalent as well as the most increasingly resistant *S. pneumoniae* serotype in invasive infections [8]. The most prevalent serotypes involved in invasive disease in Canada at the time of our patient’s presentation were (in descending order): 19A, 7F, 18C, 6A, 22F, 4, 5, 3 and 23B [9].

Given these findings, the new 13-valent vaccine (Prevnar-13®) that has recently been licensed in Canada, will likely reduce the increasingly prevalent infection rate from the 19A strain of *S. pneumoniae*. This new vaccine contains the same antigens as Prevnar-7® with six additional capsular antigens of serotypes 1, 3, 5, 6A, 7F and 19A [10], which together comprise 13 of the 91 *S. pneumoniae* serotypes described thus far [8].

## Conclusions

This case report highlights the fact that puerperal mastitis may be caused by unusual bacteria, including *S. pneumoniae.* Immunization of babies with effective pneumococcal vaccines should decrease the incidence of pneumococcal puerperal infections even further, as well as other invasive pneumococcal infections that may be similarly transmitted from baby to mother.

## Consent

Written informed consent was obtained from the patient for publication of this case report and any accompanying images. A copy of the written consent is available for review by the Editor-in-Chief of this journal.

## Competing interests

The authors declare that they have no competing interests.

## Authors’ contributions

EM performed the literature review. Both authors collected, analyzed and interpreted the clinical and microbiologic data from our patient. Both authors wrote the manuscript and read and approved the final version.
